# Inhibition of ANO1 by luteolin and its cytotoxicity in human prostate cancer PC-3 cells

**DOI:** 10.1371/journal.pone.0174935

**Published:** 2017-03-31

**Authors:** Yohan Seo, Kunhi Ryu, Jinhong Park, Dong-kyu Jeon, Sungwoo Jo, Ho K. Lee, Wan Namkung

**Affiliations:** 1 College of Pharmacy, Yonsei Institute of Pharmaceutical Sciences, Yonsei University, Incheon, Korea; 2 Department of Integrated OMICS for Biomedical Science, WCU Program of Graduate School, Yonsei University, Seoul, Korea; Albany Medical College, UNITED STATES

## Abstract

Anoctamin 1 (ANO1), a calcium-activated chloride channel, is highly amplified in prostate cancer, the most common form of cancer and leading causes of cancer death in men, and downregulation of ANO1 expression or its functional activity is known to inhibit cell proliferation, migration and invasion in prostate cancer cells. Here, we performed a cell-based screening for the identification of ANO1 inhibitors as potential anticancer therapeutic agents for prostate cancer. Screening of ~300 selected bioactive natural products revealed that luteolin is a novel potent inhibitor of ANO1. Electrophysiological studies indicated that luteolin potently inhibited ANO1 chloride channel activity in a dose-dependent manner with an IC_50_ value of 9.8 μM and luteolin did not alter intracellular calcium signaling in PC-3 prostate cancer cells. Luteolin inhibited cell proliferation and migration of PC-3 cells expressing high levels of ANO1 more potently than that of ANO1-deficient PC-3 cells. Notably, luteolin not only inhibited ANO1 channel activity, but also strongly decreased protein expression levels of ANO1. Our results suggest that downregulation of ANO1 by luteolin is a potential mechanism for the anticancer effect of luteolin.

## Introduction

ANO1, also known as transmembrane protein 16A (TMEM16A), has been identified as a calcium-activated chloride channels (CaCCs) expressed in various cell types [1–3]. ANO1 plays pivotal roles in the regulation of a wide range of biological processes, including epithelial fluid secretion, smooth muscle contraction, cell proliferation and sensory signal transduction [1, 4, 5]. In addition, ANO1 is amplified and highly expressed in a number of cancers, including prostate cancer, breast cancer, gastrointestinal stromal tumor (GIST), head-and-neck squamous cell carcinoma (HNSCC), and esophageal squamous cell carcinoma (ESCC), and involved in cancer cell proliferation, tumorigenesis and cancer progression [6–10]. Recent studies showed that pharmacological or genetic downregulation of ANO1 significantly inhibited cancer cell proliferation, migration and invasion, even though the underlying mechanisms are still uncertain [11–13]. For instance, molecular and electrophysiological studies showed strong and functional expression of ANO1 in human metastatic prostate cancer PC-3 cells, and downregulation of ANO1 expression by shRNA induced significant reduction of cell proliferation, metastasis and invasion [6]. In an orthotopic xenograft mouse model of prostate cancer using PC-3 cells, downregulation of ANO1 expression by intratumoral injection of ANO1 shRNA significantly inhibited tumor growth [6].

These results indicate that development of potent and selective small-molecule inhibitors of ANO1 may have a therapeutic potential for treatment of prostate cancer or other cancers with high levels of ANO1 expression. To date, few ANO1 inhibitors have been identified, such as CaCC_inh_-A01 (IC_50_ ~1 μM), tannic acid (IC_50_ ~6 μM), T16A_inh_-A01 (IC_50_ ~1 μM), MONNA (IC_50_ ~1 μM) and idebenone (IC_50_ ~9 μM) [13–17], and more recently we identified a novel small-molecule inhibitor of ANO1, Ani9 (IC_50_ ~77 nM), showing high potency and selectivity for ANO1 [18]. However, the mechanisms of action and pharmacological properties of these inhibitors remain unclear, and the ANO1 inhibitors are still in the early phases of the drug discovery.

Natural products have historically been a productive source of pharmaceutical leads and therapeutic drugs, and natural products and their derivatives have provided a number of cancer chemotherapeutic agents [19]. For example, natural compounds have played an essential role in the development of clinically useful anticancer agents, such as vinblastine, vincristine, topotecan, irinotecan and taxol [20], and the high structural diversity and biological efficacy of natural products make them attractive sources of novel scaffolds and drug leads for several biological targets.

Prostate cancer is the most common type of cancer and the second leading cause of cancer deaths in men, and recent studies suggest ANO1 may be a promising therapeutic target for prostate cancer [6]. In the present study, we performed a cell-based screening with a collection of natural products, a good source of novel scaffolds and drug leads for target proteins, to identify novel ANO1 inhibitors. Screening of the natural product library revealed that luteolin is a potent inhibitor of ANO1. We further investigated the effects of luteolin on cell proliferation and migration of PC-3 prostate cancer cells expressing high levels of ANO1 endogenously.

## Materials and methods

### Materials and solutions

Luteolin was purchased from Santa Cruz Biotechnology (Santa Cruz, CA) and kaempferol was purchased from Tocris Bioscience (Ellisville, MO). Chrysin, Apigenin, Galangin, and other chemicals, unless otherwise indicated, were purchased from Sigma (St. Louis, MO). The collection of bioactive natural products used for screening was prepared from the Spectrum collection of MicroSource Discovery Systems, Inc. (Gaylordsville, CT). HCO3^-^-buffered solution containing (in mM): 120 NaCl, 5 KCl, 1 MgCl_2_, 1 CaCl_2_, 10 D-glucose, 2.5 HEPES, and 25 NaHCO_3_ (pH 7.4). The half-Cl^-^ solution was prepared by replacing 65 mM NaCl with equimolar Na-gluconate in the HCO_3_^-^-buffered solution.

### Cell culture

Fisher rat thyroid (FRT) cells were stably transfected with human ANO1 (abc) or mouse ANO2 and the halide sensor YFP as described in previous study [16]. FRT cells were cultured in Coon`s modified F12 medium with 10% FBS, 2 mM L-glutamine, 100 units/ml penicillin and 100 μg/ml streptomycin at 37°C and 5% CO_2_. PC-3 cells were grown in RPMI 1640 medium supplemented with 10% FBS, 100 units/ml penicillin and 100 μg/ml streptomycin at 37°C and 5% CO_2_. There were populations of PC-3 cells with high and low expression of ANO1. To separate high and low levels of ANO1 expressing PC-3 cells, cells were stably transfected with the halide sensor YFP-H148Q/I152L/F46L and then the cells were plated on 96 well plates at 1 cell per well. After two weeks of incubation, high and low levels of ANO1 expressing PC-3 cells were separated by YFP quenching assay. ANO1 was activated by 100 μM ATP.

### Cell based screening

ANO1 and YFP expressing FRT cells were plated in 96-well black-walled microplates (Corning Inc., Corning, NY) at a density of 20,000 cells per well. Assays were done using FLUOstar Omega microplate reader (BMG Labtech, Ortenberg, Germany) and MARS Data Analysis Software (BMG Labtech) as described in our previous study [16]. Briefly, each well of 96-well plate was washed 3 times in PBS (200 μL/wash) and test compounds were added to each well at 25 μM final concentration. After 10 minutes incubation at 37°C, 96-well plates were transferred to the plate reader for fluorescence assay. Each well was assayed individually for ANO1-mediated I^-^ influx by monitoring YFP fluorescence continuously (400 ms per point) for 2 s (baseline), then 140 mM I^-^ solution containing 200 μM ATP was added at 2 s and then YFP fluorescence was recorded for 6 s. Initial iodide influx rate was determined from the initial slope of fluorescence decrease, by nonlinear regression, following infusion of iodide with ATP.

### Short-circuit current

Snapwell inserts containing ANO1-expressing FRT cells were mounted in Ussing chambers (Physiologic Instruments, San Diego, CA). The basolateral bath was filled with HCO3^-^-buffered solution and the apical bath was filled with a half-Cl^-^ solution. The basolateral membrane was permeabilized with 250 μg/mL amphotericin B. The cells were bathed for a 20 min stabilization period and aerated with 95% O_2_ / 5%CO_2_ at 37°C. Luteolin and kaempferol were applied to the apical and basolateral bath solution and then ATP was added to the apical bath solution to induce intracellular calcium increase. Apical membrane currents were measured with an EVC4000 Multi-Channel V/I Clamp (World Precision Instruments, Sarasota, FL) and recorded using PowerLab 4/35 (AD Instruments, Castle Hill, Australia). Data were collected and analyzed with ADInstruments acquisition software Labchart Pro 7 software. The sampling rate was 4 Hz.

### Intracellular calcium measurement

Intracellular calcium levels were monitored using Fluo-4 NW calcium assay kit (Invitrogen, Carlsbad, CA) as per the manufacturer's protocol in FRT and PC-3 cells. Briefly, the cells were cultured in a 96 well plate and incubated with 100 μL assay buffer including Fluo-4 NW. After 1 hour incubation, Fluo-4 fluorescence was measured with a FLUOstar Omega microplate reader equipped with syringe pumps and custom Fluo-4 excitation/emission filters (485/538 nm). Intracellular calcium increase was induced by application of 100 μM ATP.

### Patch-clamp

Patch-clamp recordings were performed on ANO1-expressing FRT cells using the whole-cell recording configuration. The bath solution contained (in mM): 140 NMDG-Cl, 1 CaCl_2_, 1 MgCl_2_, 10 glucose and 10 HEPES (pH 7.4). The pipette solution contained (in mM): 130 CsCl, 0.5 EGTA, 1 MgCl_2_, 1 Tris-ATP, and 10 HEPES (pH 7.2). Pipettes were pulled from borosilicate glass and had resistances of 3–5 MΩ after fire polishing. Seal resistances were between 3 and 10 GΩ. The liquid junction potential (~2.4 mV) was not corrected. After establishing the whole-cell configuration, whole-cell capacitance and series resistance were compensated with the amplifier circuitry. After establishing the whole-cell configuration, ANO1 was activated by ATP (100 μM). Whole-cell currents were elicited by applying hyperpolarizing and depolarizing voltage pulses from a holding potential of 0 mV to potentials between -80 mV and +80 mV in steps of 20 mV. Recordings were made at room temperature using an Axopatch-200B (Axon Instruments, Union City, CA). Currents were digitized and analyzed using a Digidata 1440A converter (Axon Instruments), and pCLAMP 10.2 software (Molecular Devices, Sunnyvale, CA). Currents were low-pass filtered at 1 kHz and sampled at 5 kHz.

### Cell proliferation assays

PC-3 cells were seeded to a confluency of ~30% on Day 1 in 96-well plates. The cells were incubated for 24 hours to reach ~50% confluence and treated with different concentrations (3, 10, 30, 100 μM) of luteolin and kaempferol, and an equal amount of DMSO was added to the all control. After 24 hour elapsed, the influence of luteolin and kaempferol on the cell viability was estimated by MTS assay. Briefly, the cells were reincubated with MTS for 1 hour. The soluble formazan produced by cellular reduction of MTS was quantified by measuring the absorbance at 490 nm with Infinite M200 (Tecan, Grödig, Austria) microplate reader. MTS assay was done using CellTiter 96 Aqueous One Solution Cell Proliferation Assay kit (Promega, Madison, WI).

### Scratch wound healing assay

PC-3 cells were cultured in a 96-well plate until they reached ~80% confluence as a monolayer and then the cell layer was wounded using a 96-Well WoundMaker (Essen BioScience, MI) and washed twice with fresh serum free media. The cells were incubated with serum free medium, and images of the wounds were taken using the IncuCyte ZOOM (Essen BioScience, MI). The wound recovery properties of test compounds were analyzed by the IncuCyte software.

### Immunoblot

Immunoblotting was performed as described previously [18]. FRT and PC-3 cells were lysed with cell lysis buffer (50 mM Tris-HCl (pH 7.4), 1% Nonidet P-40, 0.25% sodium deoxycholate, 150 mM NaCl, 1 mM EDTA, 1 mM Na_3_VO_4_, and protease inhibitor mixture). Whole cell lysates were centrifuged at 15,000 g for 10 min at 4°C to remove the cell debris, and supernatant protein was separated by 4–12% Tris-glycine precast gel (KOMA BIOTECH, Seoul, Korea) and then transferred onto PVDF membrane (Millipore, Billerica, MA). Membrane was blocked with 5% non-fat skim milk in Tris-buffered saline including 0.1% Tween 20 (TBST) for 1 hour at room temperature. The membrane was then incubated overnight with primary ANO1 antibody (1:500 dilution, ab64085; Abcam Inc., Cambridge, MA). After washing three times with TBST, the blot was further incubated for 60 min at room temperature with an anti-rabbit secondary antibody (Santa Cruz Biotechnology, CA). The membrane was then washed three times with TBST and then visualized using the ECL Plus western blotting detection system (GE Healthcare, NJ).

### Quantitative real-time PCR

Total messenger RNA (mRNA) was extracted by using a TRIzol reagent (Invitrogen, Carlsbad, CA). Quantitative Real-time PCRs were performed using the StepOnePlus Real-Time PCR System (Applied Biosystems, CA) and Thunderbird SYBR qPCR mix (Toyobo, Osaka, Japan). The thermal cycling conditions comprised an initial step at 95°C for 5 minutes followed by 40 cycles at 95°C for 10 seconds, 55°C for 20 seconds, and 72°C for 10 seconds in a 96-well reaction plate. Primers used were as follows: ANO1-sense 5′-GGA GAA GCA GCA TCT ATT TG-3′, ANO1-antisense 5′ GAT CTC ATA GAC AAT CGT GC-3′, β-actin-sense 5′-GCA AAG ACC TGT ACG CCA ACA C-3′ and β-actin-antisense 5′-ATC TCC TTC TGC ATC CTG TC-3′. ANO1 mRNA levels were normalized to β-actin levels and the fold-change in gene expression was determined by using 2^-ΔΔCT^ method. Quantitative PCR experiments were carried out in triplicates.

### Statistical analysis

The results of multiple experiments are presented as the means ± S.E. Statistical analysis was performed with Student’s t-test or by analysis of variance as appropriate. A value of *P* < 0.05 was considered statistically significant. Dose-response curves were fitted, and IC_50_ values were calculated with GraphPad Prism Software (GraphPad Software, CA).

## Results

### Identification and characterization of a novel ANO1 inhibitor, luteolin

**Fig 1:**
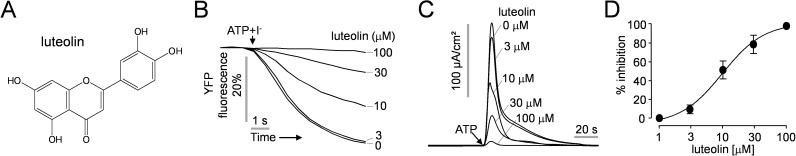
Identification of a novel inhibitor of ANO1, luteolin. A) Chemical structure of luteolin. B) ANO1 activity was measured in FRT cells expressing human ANO1 and a halide sensor YFP. ANO1 activity was inhibited by the indicated concentrations of luteolin. C) Apical membrane currents were recorded from FRT cells expressing ANO1. Representative current traces showing luteolin induced-ANO1 inhibition at the indicated concentration. ANO1 was activated by100 μM ATP. D) Summary of dose-response (mean ± S.E., n = 3–4).

**Fig 2:**
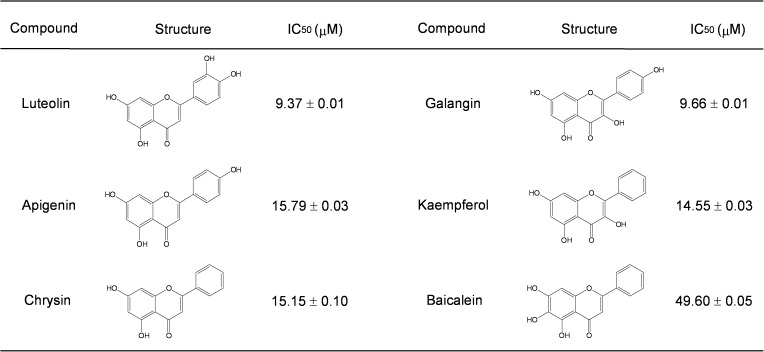
ANO1 inhibition by analogs of luteolin. IC_50_ values were determined using YFP fluorescence quenching assay in FRT cells expressing ANO1 (mean ± S.E., n = 3).

A cell-based screening of natural products was performed to identify novel ANO1 inhibitors as potential anticancer agents against prostate cancer. Effect of each natural product on ANO1 chloride channel activity was measured with YFP quenching assay in FRT cells expressing YFP-F46L/H148Q/I152L and human ANO1 (abc) as described in Method. Screening of ~300 natural products yielded 6 compounds that block iodide influx by >70% at 25 μM. As shown in Fig 1, luteolin, a flavonoid that exists in many types of plants, was identified as a bona fide ANO1 inhibitor. Luteolin potently inhibited ANO1-mediated YFP fluorescence decrease in a dose-dependent manner with IC_50_ value of 9.37 ± 0.01 μM (Fig 2) and almost completely blocked ATP-induced ANO1 activation at 100 μM (Fig 1B). Apical membrane current measurement in FRT cells expressing ANO1 showed that luteolin significantly blocked ANO1 chloride current activated by application of ATP with an IC_50_ value of 9.77 ± 0.10 μM (Fig 1C and 1D).

**Fig 3:**
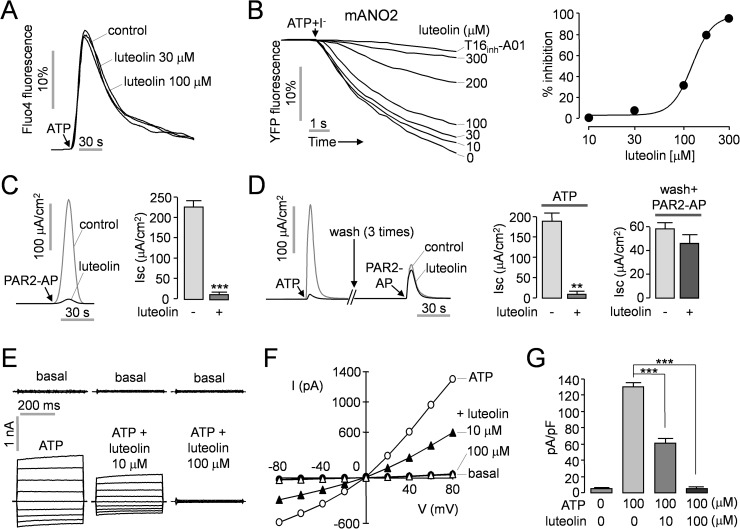
Characterization of luteolin. A) Intracellular calcium concentration was measured using Fluo-4 in FRT cells. The cells were pretreated with the indicated concentrations of luteolin for 20 min and then 100 μM ATP was applied. B) Effect of luteolin on ANO2 activity was observed in FRT cells expressing mouse ANO2 (mANO2) and a halide sensor YFP. Treatment with the indicated concentrations of luteolin inhibited ATP-induced activation of mANO2. (right) Summary of dose-response (mean ± S.E., n = 3). C) Effect of luteolin on protease-activated receptor 2 (PAR2)-induced ANO1 activation in FRT-ANO1 cells. The cells were pretreated for 20 min with luteolin (100 μM) and ANO1 was activated by PAR2 activating peptide (PAR2-AP, 50 μM). (right) Summary of peak currents (mean ± S.E., n = 3–4). D) Luteolin reversibility. After the extinction of 100 μM ATP-induced ANO1 currents, the cells were washed 3 times for 5 min each and then ANO1 was activated by 50 μM PAR2-AP. (middle, right) Summary of peak current (mean ± S.E., n = 3–4). E) Whole-cell ANO1 currents were recorded at a holding potential of 0 mV and pulsed to voltages between ± 80 mV (in steps of 20 mV) in the absence and presence of 10 μM and 100 μM luteolin in FRT-ANO1 cells. ANO1 was activated by 100 μM ATP. F) Current/voltage (I/V) plot of mean currents at the middle of each voltage pulse. G) The bar graphs summarize the current density data measured at + 80 mV (mean ± S.E., n = 5). **P < 0.01, ***P < 0.001.

Calcium signaling is essential for the regulation of ANO1 chloride channel activity. Therefore we investigated the effect of luteolin on intracellular calcium signaling using a fluorescent calcium sensor Fluo-4. FRT cells were loaded with Fluo-4 and pretreated with 30 and 100 μM luteolin for 20 minutes. As shown in Fig 3A, application of 100 μM ATP strongly increased cytosolic calcium levels and the increment of ATP-induced cytosolic calcium levels was not affected by luteolin. Anoctamin (ANO) gene family contains ten members (ANO1-ANO10), and ANO1 and ANO2, which share ~60% amino acid identity, are confirmed CaCCs [21]. To elucidate whether luteolin alters ANO2 chloride channel activity, the effect of luteolin on ANO2 activity was monitored using YFP quenching assay in FRT cells expressing mouse ANO2 (Fig 3B). ATP-induced activation of ANO2 was less potently inhibited (IC_50_ ~120 μM) by luteolin compared with that of ANO1 (IC_50_ ~9.4 μM, Fig 2).

To investigate whether luteolin reversibly inhibits ANO1, ANO1 was fully activated by application of either protease-activated receptor 2-activating peptide (PAR2-AP, 50 μM) or ATP (100 μM). PAR2-AP-triggered activation of ANO1chloride channels were almost completely blocked by 100 μM luteolin (Fig 3C). Apical membrane ANO1 currents were fully activated by both primary stimulation with PAR2-AP (Fig 3C) and ATP (Fig 3D), but secondary stimulus with PAR2-AP showed decreased ANO1 currents in Fig 3D. ATP-induced ANO1 activation was almost completely inhibited by 100 μM luteolin. However, after washing 3 times, the inhibitory effect of luteolin on PAR2-AP-induced activation of ANO1 was almost abolished (Fig 3D, right). These results suggest that ANO1 is reversibly inhibited by luteolin.

To elucidate the mechanism of action of luteolin on ANO1 chloride channels, we performed whole-cell patch clamp experiments in FRT cells expressing ANO1. In the patch-clamp experiments, ANO1 currents were recorded immediately after the intracellular calcium levels reached a maximum concentration by application of 100 μM ATP. The patch clamp measurement showed that application of luteolin blocked ATP-induced ANO1 chloride currents at all voltages, and luteolin inhibited ANO1 chloride currents by 55.3 ± 2.8% and 99.2 ± 0.2% at 10 and 100 μM, respectively (Fig 3E–3G).

We also investigated the effect of other flavonoids on ANO1 activity using YFP quenching assay in FRT cells expressing YFP and ANO1. The inhibitory effects of apigenin, chrysin, kaempferol, baicalein and galangin on ANO1 activity were summarized in Fig 2.

### Luteolin inhibits PC-3 cell proliferation and wound healing

**Fig 4:**
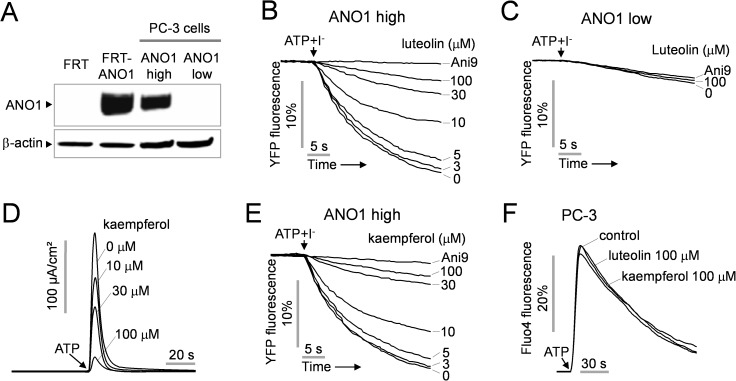
Effect of luteolin and kaempferol on the ANO1 activity of PC-3 cells. A) Immunoblot of ANO1 protein in FRT, FRT-ANO1 and PC-3 cells expressing high and low levels of ANO1. Representatives of three sets of studies are shown. B, C) ANO1 activity was measured in PC-3 cells expressing high and low levels of ANO1. ANO1 activity was inhibited by the indicated concentrations of luteolin and Ani9 (1 μM), a specific ANO1 inhibitor. D) Effect of kaempferol on ANO1 channel activity was observed in FRT-ANO1 cells. Indicated concentration of kaempferol was pretreated for 20 min and then 100 μM ATP was applied to activate ANO1. E) Effect of kaempferol on ANO1 activity in PC-3 cells expressing high levels of ANO1. F) Effect of luteolin and kaempferol on calcium signaling was measured using Fluo-4 in PC-3 cells.

ANO1 is the major CaCC in PC-3 cells and inhibition of ANO1 significantly decreases cell proliferation in human metastatic prostate cancer PC-3 cells [6]. In this regard, we examined the effect of luteolin on prostate cancer cell proliferation using PC-3 cells. In Fig 4A, immunoblotting shows high and low levels of ANO1 expressing PC-3 cells separated by YFP quenching assay as described in Methods. To investigate the effect of luteolin on ANO1 activity in prostate cancer cells, YFP quenching assay was performed in high and low levels of ANO1 expressing PC-3 cells. As shown in Fig 4B, ATP-induced ANO1 activation strongly decreased YFP fluorescence and the fluorescence decrease was completely blocked by Ani9, an ANO1 specific inhibitor. Luteolin potently inhibited ANO1 activity in a dose-dependent manner in PC-3 cells expressing high levels of ANO1, while there was no ANO1 activity detectable in PC-3 cells with low levels of ANO1 expression (Fig 4C).

Kaempferol, an analogue of luteolin, potently blocked ANO1 chloride channel current by 36.2 ± 3.0%, 55.0 ± 4.1% and 89.3 ± 2.0% at 10, 30 and 100 μM, respectively, in ANO1 expressing FRT cells (Fig 4D). Kaempferol also significantly inhibited ANO1 activity in PC-3 cells expressing high levels of ANO1 (Fig 4E), and both luteolin and kaempferol did not alter ATP-induced intracellular calcium increase in PC-3 cells (Fig 4F).

**Fig 5:**
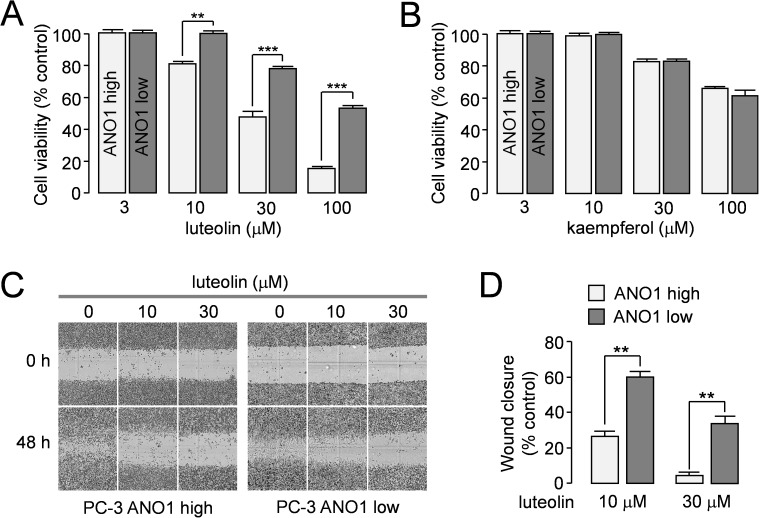
Effect of luteolin and kaempferol on the cell viability and migration of PC-3 cells. A, B) PC-3 cells expressing high and low levels of ANO1 were treated with luteolin and kaempferol at the indicated concentration, and cell proliferation was measured after 24 hour incubation using MTS assay (mean ± S.E., n = 6). C, D) Wound healing assay was performed on PC-3 cells expressing high and low levels of ANO1. The cells were treated with 10 and 30 μM luteolin, and representative images were taken at 0 h and 48 h post wounding (× 10). (right) The wound closure was quantified at 48 h post-wound (mean ± S.E., n = 5). **P < 0.01, ***P < 0.001.

Interestingly, inhibitory effect of luteolin on cell proliferation was significantly stronger in PC-3 cells with high levels of ANO1 expression than PC-3 cells with low levels of ANO1 expression (Fig 5A). However, inhibition of cell proliferation by kaempferol did not show the significant difference between high and low levels of ANO1 expressing PC-3 cells (Fig 5B). To observe the effect of ANO1 inhibition by luteolin on cell migration, wound healing assay was performed in PC-3 cells expressing high or low levels of ANO1 (Fig 5C). Wound recovery was more strongly inhibited by luteolin in PC-3 cells expressing high levels of ANO1 compared with PC-3 cells expressing low levels of ANO1 (Fig 5D).

### Luteolin significantly decreases protein levels of ANO1 in PC-3 cells

**Fig 6:**
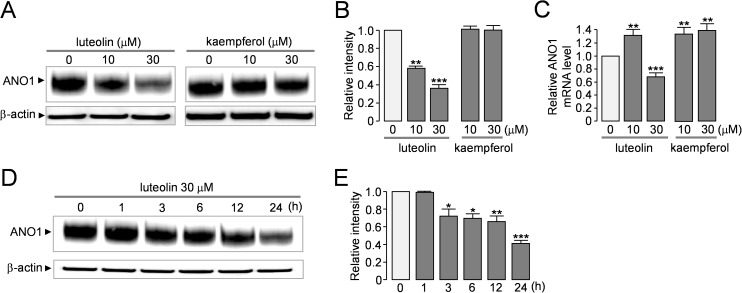
Effect of luteolin and kaempferol on the expression levels of ANO1 in PC-3 cells. A) Immunoblot analysis of ANO1 in PC-3 cells expressing high levels of ANO1. Cells were incubated with indicated concentration of luteolin and kaempferol for 24 hour. B) The ANO1 band intensity was normalized to β-actin (mean ± S.E., n = 3–4). C) Effect of luteolin and kaempferol on gene expression levels of ANO1 in the PC-3 cells (mean ± S.E., n = 3). D) Luteolin-induced time-dependent changes of ANO1 protein levels in the PC-3 cells. E) The ANO1 band intensity was normalized to β-actin (mean ± S.E., n = 3). *P < 0.05, **P < 0.01, ***P < 0.001.

To elucidate the different effects of luteolin and kaempferol on the proliferation of PC-3 cells expressing high and low levels of ANO1, we observed the effect of luteolin and kaempferol on protein and mRNA expression levels of ANO1. As shown in Fig 6A and 6B, luteolin significantly decreased protein levels of ANO1 by 41.2 ± 1.5% and 63.1 ± 5.7% at 10 and 30 μM, respectively, in PC-3 cells expressing high levels of ANO1. However, kaempferol did not affect the ANO1 protein levels. Quantitative real time PCR revealed that the gene expression levels of ANO1 were slightly increased by 10 μM luteolin and kaempferol but decreased by 30 μM luteolin (Fig 6C). To investigate luteolin-induced time-dependent changes of ANO1 protein levels, the PC-3 cells were treated with 30 μM luteolin and then changes of ANO1 protein levels were determined by immunoblotting analysis (Fig 6D). As shown in Fig 6E, ANO1 protein levels were significantly decreased in a time-dependent manner after treatment with luteolin.

## Discussion

ANO1/TMEM16A, a calcium-activated chloride channel (CaCC), has been shown to be highly amplified in several human cancers and recent evidence suggests ANO1 as a potential therapeutic target for cancer because down regulation of ANO1 reveals therapeutic advantages on HNSCC, breast, prostate and lung cancer treatment [6, 8, 9, 22]. In the present study, we performed a cell-based screening to identify novel ANO1 inhibitor from natural products and revealed luteolin as a bona fide inhibitor of ANO1. Electrophysiological studies showed that luteolin almost completely inhibited ANO1 chloride channel activity in a dose-dependent manner without affecting intracellular calcium signaling (Figs 1C, 2A and 2E–2G). In addition, application of 10 μM luteolin significantly decreased protein expression levels of ANO1 but not mRNA levels in PC-3 cells (Fig 6A–6C). These results suggest the possibility that luteolin might directly bind to ANO1 and decrease protein stability of ANO1 chloride channels.

Previous studies have shown that pharmacological inhibition of ANO1 by small-molecule ANO1 inhibitors such as T16A_inh_-A01, CaCC_inh_-A01, tannic acid, MONNA and idebenone significantly decreased cell proliferation, migration or invasion abilities in human cancer cell lines expressing ANO1, even though the pathophysiological roles of ANO1 in cancer and the underlying molecular mechanisms of anticancer effect of downregulation of ANO1 are not clearly understood [12, 13, 23, 24]. Interestingly, as shown in Fig 5A and 5C, luteolin inhibited cell proliferation and migration of PC-3 human prostate cancer cells expressing high levels of ANO1 much more potently than that of ANO1-deficient PC-3 cells. However, kaempferol, an analogue of luteolin which showed potent inhibition of ANO1 channel activity (Fig 4D and 4E), did not show any difference in cell proliferation between the PC-3 cells expressing high and low levels of ANO1(Fig 5B). In addition, luteolin inhibited cell proliferation of PC-3 cells expressing high levels of ANO1 more potently than kaempferol (Fig 5A and 5B). The different effects of luteolin and kaempferol on PC-3 cells expressing high and low levels of ANO1 may be due to the distinct effects of these compounds on protein stability of ANO1 as shown in Fig 6. Luteolin inhibits both ANO1 channel activity and protein stability but kaempferol inhibits only ANO1 channel activity. Downregulation of ANO1 expression will have more efficient anticancer effects compared with ANO1 inhibitors because it reduces both the interaction of ANO1 with other proteins involved in cell growth regulation and the chloride channel activities affecting cell proliferation and migration. Thus we believe that the dual effect of luteolin on protein levels and channel activities of ANO1 may be more beneficial for the downregulation of ANO1 in cancer cells.

Luteolin, a common dietary flavonoid, is abundant in vegetables such as cabbage, celery, and broccoli, and has various biological effects including anticancer activity against multiple types of human cancer cell lines [25]. In prostate cancer, luteolin exerts anticancer effects through various mechanisms such as suppression of androgen receptor expression and vascular endothelial growth factor receptor 2 (VEGFR-2) mediated angiogenesis at > 10 μM, and induction of cytotoxicity, apoptosis and cell cycle arrest via regulation of IGF-1/Akt pathway, Akt-Mdm2 pathway, epidermal growth factor signaling pathway and expression levels of miR-630 and miR-301 at > 10 μM [26–30]. ANO1 is highly expressed in prostate cancer tissues and downregulation of ANO1 results in reduction of proliferation, progression and pathogenesis of metastatic prostate cancer cells [6]. In addition, intraperitoneal application of 10 mg/kg and 50 mg/kg of luteolin significantly decreased the volume and the weight of solid tumors in xenograft mouse model of PC-3 and LNCaP prostate cancer, respectively [26, 28]. Interestingly, free form of luteolin is detected in rat plasma. Oral administration of 50 μmol/kg (~14.3 mg/kg) of luteolin in propyleneglycol reached a maximum level ~3.08 μM and total luteolin containing free luteolin and its conjugates reached a maximum level ~15.5 μM [31]. Here, we showed that luteolin is a potent inhibitor of ANO1 (IC_50_ ~10 μM) and blocks cell proliferation and migration of PC-3 cells expressing high levels of ANO1 much more strongly than that of ANO1-deficient PC-3 cells. Collectively these evidences support a possible novel mechanism underlying the anticancer effects of luteolin against prostate cancer cells via downregulation of ANO1.

As shown in Fig 6C, mRNA expression levels of ANO1 were slightly (~1.3 fold) but significantly increased by treatment with luteolin (10 μM) in PC-3 cells. However, interestingly, application of 30 μM luteolin significantly decreased the ANO1 gene expression levels compared with that of control. In Fig 5A, application of 30 μM luteolin strongly decreased cell viability of PC-3 cells expressing high levels of ANO1 by 52.6 ± 3.8%. Thus the cytotoxicity of luteolin at high concentration may affect ANO1 gene expression. In addition, previous studies suggest that luteolin may regulate gene expression via type II [^3^H]estradiol binding site in PC-3 cells [32, 33].

In summary, a novel ANO1 inhibitor, luteolin, potently inhibited ANO1 chloride channel activity without affecting intracellular calcium signaling. Interestingly, luteolin significantly decreased not only ANO1 channel activity but also protein stability of ANO1. In addition, luteolin reduced cell proliferation and migration of PC-3 cells expressing high levels of ANO1 much more potently than that of ANO1-deficient PC-3 cells. These results indicate that downregulation of ANO1 by luteolin is a novel mechanism underlying the anticancer effect of luteolin on various human cancers including prostate cancer.
